# A prospective diary study of chronic pain-related intrusive mental imagery: “trolls climbing up my spine with ice axes” and other vivid images

**DOI:** 10.1097/j.pain.0000000000004009

**Published:** 2026-05-29

**Authors:** Luzia Grabherr, Daniel Ferreiro Couto, Carlo Delli Noci, Marc R. Suter, Antje Horsch, Chantal Berna

**Affiliations:** a Center for Integrative and Complementary Medicine, Department of Anesthesiology, Lausanne University Hospital (CHUV), and University of Lausanne, Lausanne, Switzerland; b The Sense Innovation and Research Center, Lausanne and Sion, Switzerland; c Liaison Psychiatry Service, Department of Psychiatry, Lausanne University Hospital (CHUV), Lausanne, Switzerland; d Pain Center, Department of Anesthesiology, Lausanne University Hospital (CHUV), Lausanne, Switzerland; e Service of Neonatology, Department Woman-Mother-Child, Lausanne University Hospital (CHUV), Lausanne, Switzerland; f Institute of Higher Education and Research in Healthcare, University of Lausanne, Lausanne, Switzerland

**Keywords:** Cognition, Mental imagery, Chronic pain, Cognitive behavioral therapy, Diary study

## Abstract

People with chronic pain can experience intrusive mental images (IMI) related to pain, that is, cognitions in the form of sensory-like experiences without actual sensory input. Previous retrospective studies suggested that such images are prevalent, associated with strong emotions, and contribute to pain maintenance. We prospectively investigated pain-related IMI occurrence and characteristics in patients with chronic secondary postsurgical or posttraumatic pain using electronic diaries, aiming to examine factors associated with pain reported after IMI. Of the 50 study completers, 35 (70%) reported pain-related IMI. They scored significantly higher on the Spontaneous Use of Imagery Scale (*t* = −2.93, *P* < 0.001), PTSD Checklist for DSM-5 (*t* = −3.98, *P* < 0.0001), and Hospital Anxiety and Depression Scale depression score (*t* = −2.11, *P* = 0.045) than those without IMI. On average, patients experiencing IMI reported 6.5 images/week, with moderate vividness (mean 6.5/10), emotional intensity (mean 5.4/10), and pain after IMI (mean 6.0/10). Intrusive mental images were mostly of negative valence (81.6%). However, almost one-third reported coping images. Baseline pain measures correlated with IMI vividness, emotional intensity, and valence but not image frequency. Multilevel modelling indicated that IMI vividness, emotional intensity, and valence were significant predictors of postimage pain, with pain catastrophizing also emerging as a significant baseline predictor. Hence, IMI may interact with pain experiences through IMI characteristics. This prospective study highlights a high prevalence and frequent occurrence of IMI in chronic secondary pain and associations with pain following imagery episodes. These findings underscore a possible role for imagery-targeted therapeutic interventions.

## Abstract 2

Supplemental Digital Content is Available in the Text.

In a prospective diary study capturing a high prevalence of intrusive mental imagery in chronic secondary pain, imagery vividness, emotional intensity, and valence were linked to postimage pain.

## 1. Introduction

Mental images are nonverbal cognitions that can be experienced in different sensory modalities (visual, auditory, olfactory, gustatory, cutaneous, kinesthetic) in the absence of their respective sensory input.^39,49^ Mental images are known to evoke stronger emotions than verbal cognitions.^33,42^ They can be generated voluntarily or experienced intrusively.^13^

Intrusive mental images (IMI) are of particular interest in research and clinically, due to the key role they can play in psychopathology, particularly in posttraumatic stress disorder (PTSD).^18^ However, IMI are relevant to numerous other disorders,^13,26,35^ including anxiety disorders such as social phobia^25,30,57^ and mood disorders.^32,45^ For example, in major depressive disorder, IMI can represent traumatic memories related to the event that triggered a depressive episode. Similar to flashbacks in PTSD, these images can evoke a sensation of reliving the depressive event and are associated with significant emotional distress.^48^ Consequently, IMI are believed to have an influence on the development and/or the maintenance of various pathologies and represent a potential therapeutic target.^13,37^

Recent retrospective studies show that people with chronic pain can also experience pain-related IMI, with a prevalence varying from 23% to 100%.^5,14,21–23,51,66,69^ The reported frequency also varies largely, with 18% to 81% of participants experiencing them daily.^22,23,69^ These images can be recurrent.^51^ Furthermore, when IMI are re-evoked voluntarily, patients report increased pain and negative emotions and decreased positive emotions.^5,51^ Patients experiencing IMI also reported a moderate to severe interference with daily life^22^ and avoidance of activities^67^ along with higher levels of anxiety and depressive symptoms than those without IMI.^21,22^ Models based on cognitive behavioral therapy (CBT) frameworks have been proposed, where images can contribute to a negative vicious cycle, reinforcing negative emotions, avoidance, and pain.^4,5,68^

IMI can evolve with patients modifying them spontaneously to make them less distressing,^5,21^ or with the help of imagery-targeting therapy. In fact, different therapies include mental imagery to address chronic pain, including art therapy,^14,52^ guided imagery,^36,53^ hypnosis,^14,65^ imagery rescripting,^31,50^ eye movement desensitization and reprocessing (EMDR),^24,46,61^ and graded motor imagery.^12^

In contrast to this knowledge, research investigating IMI in chronic pain remains relatively scarce. It relies on retrospective reports (interviews or postal surveys), except for 1 recent investigation using ecological momentary assessments (EMAs).^67^ However, retrospective studies are susceptible to biases, including recall errors or overestimation of negative mood.^60^ Therefore, this study used an electronic diary coupled with interviews to collect prospective data during 1 week in patients with chronic secondary pain, studying the occurrence and characteristics of IMI in a naturalistic setting. The aims of this study were to examine differences in baseline pain and psychological measures between those with and without IMI, as well as to assess whether specific IMI characteristics and baseline psychological measures predicted pain intensity after the IMI.

## 2. Methods

### 2.1. Participants

Participants were recruited at the academic Pain Center of the Lausanne University Hospital. Adult patients (≥18 years) with secondary chronic pain (>6 months) meeting ICD-11 criteria for chronic postsurgical or posttraumatic pain (MG30.2) were eligible if they were able to communicate in French without a translator, owned a smartphone or tablet, and had no acute or severe psychiatric condition (eg, suicidal ideation or active PTSD). Pain physicians identified potential patients and offered them the possibility to participate in this study. If patients agreed to be contacted, an investigator presented detailed information either by telephone or face-to-face and provided them with the consent form. Eligible patients expressing interest were invited to attend the first interview at the Pain Center.

The aim was to collect 35 completed diaries of participants experiencing IMI related to their chronic pain. This target number of participants with intrusions was based on studies examining intrusions using electronic diaries in the context of PTSD.^38,56^ As the prevalence was unknown, recruitment continued until this set number of diary participants was reached.

### 2.2. Ethics

This study was approved by the local ethics committee (CER-VD 2017-01132) and was conducted in accordance with the Declaration of Helsinki. All participants provided written informed consent during the first study visit. Participants were reimbursed for their travel expenses.

### 2.3. Procedure

An overview of the procedure is shown in supplemental digital content, Figure 1 (http://links.lww.com/PAIN/C505).

#### 2.3.1. First interview

This interview allowed for informed consent and verification of inclusion/exclusion criteria.

Next, the following participant characteristics were collected: age, sex, medication (by category: anti-inflammatory; paracetamol; opiates; antidepressants; etc), event that led to chronic pain (by category: accident or surgery), duration of chronic pain, number of pain sites (ie, the total count of distinct anatomical locations reported as painful; eg, knee pain and lower back pain = 2 sites), and comorbidity.

Then, the presence of pain-related IMI was explored through a structured interview that was based on prior work.^5^ IMI were explained to the participants and defined as nonverbal thoughts that are experienced in a sensory modality (visual, auditory, olfactory, gustatory, cutaneous, and/or kinesthetic) but without peripheral sensory input. Two general, nonpain-related examples were provided: (1) seeing the image of a relative's face in your mind's eye and (2) playing your favorite song in your mind. Then, the notion of intrusiveness was clarified as “an involuntary experience.” After explaining that such IMI can also occur in relation to pain, participants were asked whether they were familiar with this. When the participant indicated not having pain-related IMI, examples related to chronic pain were provided (eg, involuntarily imagining a sharp object pressing into the pain site). Some participants became aware of experiencing IMI when given such more precise examples. Participants who reported IMI were asked to provide descriptions to ensure their experience fulfilled our definition. Particular attention was paid to distinguish IMI from metaphors. Pain metaphors are descriptions that aim to enhance comprehension/communication and empathy in an interpersonal context.^2,27,28^ For example, a person suffering from pain could describe the sensation to another person “like fire” without experiencing any sensory mental images (ie, no image of a fire in their mind).

#### 2.3.2. Online questionnaires

After the interview, a link was sent to all participants' email addresses or mobile phone numbers to access the online questionnaires (see 2.4.1 section). Those without Internet access were provided with a printed version. The questionnaires and diary were built with Sphinx (Le Sphinx Développement, France) and hosted on a secure server at Lausanne University. A randomly generated personal code allowed the investigators to link the answers to the participant. If the participant failed to complete the questionnaires, reminders were sent through telephone calls or text messages. An investigator assisted by phone the few participants who were struggling to complete the questionnaires themselves.

The participation of patients who did not report pain-related IMI during the baseline interview ended after completion of the online questionnaires.

Those who reported at least 1 IMI related to their pain per week during the previous 2 weeks were given instructions about the electronic IMI diary and the second interview (debriefing).

#### 2.3.3. Diary

Participants received an email summarizing the instructions, which included both a link and a QR code for accessing the IMI diary (either of which could be used). They then received an automated reminder email every morning. They were instructed to immediately access the diary as soon as an IMI came to mind, and to do so as many times as they had IMI during the day. If immediate reporting was not feasible, they were asked to do so as soon as possible. It was emphasized that the report should be systematic, even if the experienced image was a recurrent one. Nevertheless, if frequent images made reporting impractical, they could report a manageable number while tracking the unreported ones. All images, even ones that were not discussed in the first interview, were to be reported.

On the last day, an email was sent with a link to a brief final questionnaire. It included a re-assessment of the Hospital Anxiety and Depression Scale and evaluated whether the participant felt the IMI frequency was typical during the observation period (by category: much more frequently, a little more frequently, at the same frequency, a little less frequently, much less frequently *than usual*). This allowed to assess the potential impact of keeping the diary (as it might have acted as an image trigger).

During the diary-keeping period, participants were invited to contact the investigator for any questions or if a technical issue or psychological distress arose. In the case of psychological distress, the severity was to be assessed by a study clinician, and if necessary, additional support would be offered.

#### 2.3.4. Second interview (debriefing)

The second structured interview was scheduled anytime from the day the diary was completed up to 10 days afterward. It was conducted by an experienced clinician. The interview allowed for assessing the thematic categories (see 2.4.2 section) and correcting inconsistent data or unclear reports. For example, doubts about whether a reported image matched the definition of a pain-related IMI were clarified, images suggesting unreported modalities (eg, “a red balloon inflated in the leg” with no visual modality mentioned) were further explored, and apparent contradictions, such as mismatches between the reported emotion (eg, fear) and valence (eg, positive), were addressed. If time allowed, participants were also asked during the debriefing whether they had spontaneously or voluntarily modified any IMI, based on prior reports of such modified images.^5,21^ The interview also investigated potential missing data from the diary. Reasons for nonreplies were explored, and, if possible, certain data points were completed (eg, based on personal pain diaries). Patients were asked whether they perceived a relationship between pain and IMI (ie, IMI triggering pain or pain triggering IMI). Finally, this interview was an opportunity to answer the participants' questions and to provide feedback.

### 2.4. Main measures

#### 2.4.1. Baseline questionnaires

All participants completed the validated French version^55^ of the Brief Pain Inventory (BPI)^15^ which assesses *pain severity* on 4 items (worst pain over the past 24 hours, least pain over the past 24 hours, pain on average, and right now) on a numeric rating scale (NRS) from 0 (no pain) to 10 (worst pain imaginable) as well as the *interference of pain* on 7 aspects of daily life (general activity, mood, walking ability, normal work, etc.) also on a NRS from 0 (did not interfere) to 10 (interfered completely). The BPI is widely used and has demonstrated good psychometric qualities.^14,54^ In the current sample, it demonstrated good internal consistency for pain severity (Cronbach α = 0.880) and for pain interference (Cronbach α = 0.842).

Anxiety and depression symptoms were assessed with the Hospital Anxiety and Depression Scale (HADS), a 14-item self-reporting questionnaire comprising 2 subscales (anxiety and depression). Items are rated on a 4-point Likert-type scale, with higher scores suggesting greater severity of anxiety and depressive symptoms. The HADS has demonstrated good internal consistency and construct validity and is widely used to assess symptoms in medical and research settings.^7,11,74^ Nevertheless, the 2-factor structure has been questioned,^16^ which is why the HADS-Total score is used as the main measure. In the current sample, the questionnaire demonstrated acceptable internal consistency for anxiety (Cronbach α = 0.720) and good internal consistency for depression (Cronbach α = 0.831) and the HADS-Total score (Cronbach α = 0.848).

Pain-related catastrophic thinking was assessed with the Pain Catastrophizing Scale (PCS), a 13-item self-report questionnaire evaluating verbal negative pain-related cognitions comprising 3 subscales (magnification, rumination, and helplessness). Items are rated on a 5-point Likert-type scale, with higher scores suggesting a higher occurrence of catastrophic pain-related thoughts. The PCS has demonstrated strong internal consistency and construct validity.^20,64^ Internal consistency was also very strong in this study (Cronbach α = 0.932).

Posttraumatic stress disorder symptoms, such as intrusions, avoidance, negative alterations in cognitions and mood, and alterations in arousal/reactivity, were assessed with the PTSD Checklist for DSM-5 (PCL-5), a 20-item self-report measure designed to evaluate the severity of these symptoms. Each item is rated on a 5-point Likert scale ranging from 0 (not at all) to 4 (extremely), with higher scores indicating greater PTSD symptom severity. The PCL-5 has shown strong psychometric properties, making it a reliable and valid tool for assessing PTSD symptoms in both clinical and research contexts.^1,10^ Internal consistency was very strong in this study (Cronbach α = 0.921).

The level of spontaneous use of visual imagery in everyday life situations was assessed with the Spontaneous Use of Imagery Scale (SUIS), a 12-item self-report questionnaire. Items such as “If I am looking for new furniture in a store, I always visualize what the furniture would look like in particular places in my home” are rated from 1 (never appropriate) to 5 (always completely appropriate). The SUIS has demonstrated good internal consistency and construct validity.^44,58^ The French version developed by Favrod and colleagues,^19^ based on a forward-backward translation and cultural adaptation with a Cronbach alpha of α = 0.802 was used in the absence of a validated version. This study demonstrated good internal consistency of the SUIS (Cronbach α = 0.860).

#### 2.4.2. Intrusive mental image characteristics

The diary collected 1 report per pain-related IMI (allowing calculation of daily and weekly IMI frequency) with the following details (see the full diary instructions in the supplemental digital content, materials, Table 1, http://links.lww.com/PAIN/C505): *brief description of the content* (keywords), *recurrence* (categorical: new image or recurrent image), description of *the context and potential trigger* of the intrusion (keywords with 5 potential options based on previous research^5^ were suggested: pain, discussion, movement, memory, other), assessment of *intrusiveness* and *vividness* on visual analogue scales (VAS; with anchors of “no intention” and “image voluntarily evoked,” respectively, “not vivid” and “extremely vivid”), *predominant associated emotion* (categorical: sadness, fear, anger, relief, calm, other), *intensity of the predominant emotion* (on a VAS scale with anchors of “no emotion felt” and “extremely strong”), *valence of the predominant emotion* (on a VAS scale with anchors of “extremely unpleasant” and “extremely pleasant”), and *sensory modality* (categorical with multiple responses possible: visual, auditory, gustatory, olfactory, cutaneous, kinesthetic). After describing an IMI in the diary, participants had to rate their pain intensity (“postimage pain”) on a VAS consisting of a continuous horizontal line with anchors at each end: “no pain” and “worst imaginable pain.” Visual analogue scale responses were scored from 0 to 10; except for valence, which was scored from −10 to 10.

Unfortunately, the question meant to assess intrusiveness was poorly formulated: “Intrusiveness: how much did you intend to have this image from ‘no intention’ to ‘image voluntarily evoked’?” This phrasing inadvertently created a reverse scale between the title and the question (ie, an answer to the question being “very intrusive” would have to be transposed to “no intention”, so “low” on the proposed scale) causing confusion. As a result, these data were considered unreliable and were not analyzed.

##### 2.4.2.1. Thematic categories

Prior research^2,5,22,51,72^ suggests that the contents of pain-related IMI can be categorized into 8 different image categories: (1) the pain itself either in the form of an anatomical or metaphorical representation, (2) oneself in pain, (3) social interactions in the context of pain, (4) the future related to pain, (5) memories of a pain-related event, (6) aiming to cope, (7) negative images of oneself in the context of pain, and (8) abstract images related to pain.

These 8 categories were presented to participants, with the additional option of “other.” Each image could be attributed to multiple categories. Participants categorized the reported images during the last interview. In general, the attributions made by the participants corresponded to the investigators' assessment. However, on occasion, participants assigned the image to an unexpected category. In some cases, this was justified by the participant (eg, image of “being pricked by needles” was classified as a positive coping image as it helped calm the pain). Sometimes, the discrepancy was due to a misunderstanding of the proposed categories, which allowed for correction by the participant.

### 2.5. Data analysis

Patients with more than 20% missing data in the baseline questionnaires were considered unreliable and excluded from the analysis (n = 1, in the “without IMI” group).

Differences between patients with and without IMI were assessed using chi-square (χ^2^) tests for categorical variables (sex; event leading to chronic secondary pain: postsurgical or posttraumatic). The normality of continuous variables was assessed prior to analysis using visual inspection and the Shapiro-Wilk test. Depending on the distribution, group differences were analyzed using either unpaired 2-tailed t-tests assuming heteroscedasticity or the Mann-Whitney *U* test for nonnormally distributed variables (ie, pain duration and pain sites). Paired two-tailed t-tests were used to assess differences in psychometric tests within patients with IMI before and after keeping a diary (HADS scores). One-way ANOVAs were conducted to assess differences in postimage pain, emotional intensity, and valence across image categories (ie, the pain and IMI characteristics scores were averaged for their given category). Based on a Levene test, Welch's ANOVA was used to account for unequal variances when needed. To further analyze significant results, post hoc pairwise comparisons were conducted applying a Bonferroni correction. Correlations between baseline psychological measures (HADS, PCS, PCL-5, and SUIS), BPI pain scores (severity and interference), participant-level frequency of IMI, and participant-level averages of IMI characteristics (vividness, emotional intensity, and valence), and postimage pain averages were assessed using 2-tailed Spearman correlations. These statistical analyses were performed using IBM SPSS Statistics, version 29.0 (IBM Corp.).

Finally, and in line with Todd et al.,^67^ multilevel modelling was used to examine within-person diary characteristics and between-person baseline psychological measures as predictors of post-IMI pain. These analyses were conducted in R (version 4.5.2; R Foundation for Statistical Computing) using RStudio. Participants who completed at least 1 diary entry were included. Of the 33 participants contributing diary data, 2 had incomplete data and were excluded from the multilevel analyses, resulting in a final sample of 205 diary entries from 31 participants. Given the nested structure of the data (repeated diary entries within individuals), linear mixed-effects models with random intercepts for ParticipantID were estimated using the lme4 package. An intercept-only model was first fitted to estimate the intraclass correlation coefficient (ICC), quantifying the proportion of variance attributable to between-person differences. Diary-level imagery characteristics (vividness, emotional intensity, and valence) were specified as level-1 predictors and were person-mean centered. Baseline psychological variables (HADS-Total, PCS, PCL-5) and trait imagery use (SUIS) were included as level-2 predictors. Final model estimates were obtained using restricted maximum likelihood estimation with Satterthwaite approximations for degrees of freedom using the lmerTest package. Semi-partial (partial) R^2^ values were calculated to quantify the unique contribution of each predictor. Multicollinearity among predictors was assessed using variance inflation factors (VIFs) calculated with the car package in R.

## 3. Results

### 3.1. Participants

**Figure 1.**
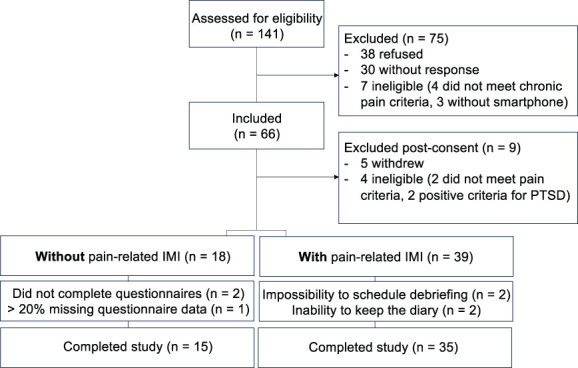
Flow diagram of the recruitment and study progress.

Sixty-six participants gave consent to take part in this study (see flow chart in Fig. 1). Of those, 9 were excluded (5 withdrew from this study, 2 did not meet chronic pain criteria, 2 met criteria for posttraumatic stress disorder and were offered a referral to a mental health specialist at the Pain Center).

During the first interview, 39 of the 57 participants reported at least 1 pain-related IMI per week over the 2 preceding weeks and were included in the diary phase of this study. Of the 18 patients without IMI, 2 participants did not complete the questionnaires despite reminders, and 1 participant returned an incomplete questionnaire (>20% missing data) and was excluded, leaving 15 participants without IMI for analyses.

Of the 39 patients invited to participate in the diary, 4 discontinued participation (2 due to the impossibility of scheduling a debriefing, 2 due to the inability to keep the diary), leaving 35 with IMI for analyses. No participants manifested distress related to journal keeping. Prevalence of IMI in the study completers was hence 35/50 (70%).

Table 1 illustrates the main characteristics of participants, comparing those with and without IMI. Participants in both groups were comparable in age (mean age of the full sample = 45.8 years), sex distribution (68% women in the full sample), distribution of chronic secondary pain type (54% postsurgical vs 46% posttraumatic in the full sample), pain duration (median of the full sample = 42 months), number of pain sites (median in the full sample = 1 pain site), BPI pain scores (BPI mean severity in the full sample = 5.0 and BPI mean interference = 4.5), pain catastrophizing (PCS; mean score in the full sample = 23.6), HADS-Total (mean score in the full sample = 15.1), and anxiety (HADS-A; mean score in the full sample = 8.6). However, the IMI group had significantly higher scores on the depression subscale of the HADS, the PCL-5, and the SUIS (Table 1).

**Table 1:** Comparison of demographic, pain, and psychological measures between participants with and without pain-related intrusive mental images (IMI).

Variable	Without IMI	With IMI	*t (df)/χ2 (df)/U*	*P*
	n = 15	n = 35		
	M (SD)/%/Md (Q1-Q3)	M (SD)/%/Md (Q1-Q3)		
Age	47.8 (11.7)	44.9 (11.1)	0.81 (25.4)	0.42
Sex (f)	73.3%	65.7%	0.28 (1)	0.60
Event (accident)	53.3%	54.3%	0.00 (1)	0.95
Pain duration (mos)	54.0 (32-89)	41.0 (20-87)	237	0.59
Pain sites (n)	1.0 (1-2)	1.0 (1-2)	269	0.87
BPI severity	4.5 (1.9)	5.4 (1.8)	−1.56 (25.2)	0.13
BPI worst pain	5.7 (2.9)	7.0 (1.6)	−1.61 (18.0)	0.13
BPI least pain	2.9 (2.1)	4.1 (2.3)	−1.79 (29.4)	0.08
BPI on average	5.1 (2.4)	5.3 (2.1)	−0.25 (21.7)	0.81
BPI pain right now	4.4 (2.6)	5.4 (2.3)	−1.31 (23.8)	0.20
BPI interference	3.7 (2.3)	5.0 (1.9)	−1.91 (23.4)	0.07
HADS-Total	12.4 (7.3)	16.3 (6.3)	−1.80 (23.6)	0.09
HADS-A	8.0 (3.9)	9.1 (3.3)	−0.93 (23.1)	0.36
HADS-D	4.4 (4.4)	7.2 (4.1)	−2.11 (25.5)	**0.045**
PCS	22.1 (12.3)	24.2 (12.6)	−0.56 (27.1)	0.58
PCL-5	11.7 (10.3)	25.7 (13.5)	−3.98 (34.5)	**<0.001**
SUIS	26.3 (17.6)	40.3 (9.2)	−2.93 (17.3)	**<0.01**

Statistically significant *P*-values (*P* < 0.05) are shown in bold.

Md, median, Q1‐Q3, interquartile range (concerns pain duration and pain sites).

BPI, Brief Pain Inventory; HADS, Hospital Anxiety and Depression Scale; PCS, Pain Catastrophizing Scale; PCL-5, PTSD Checklist for DSM-5; SUIS, Spontaneous Use of Imagery Scale.

### 3.2. Intrusive mental image characteristics

Participants reported a total of 226 pain-related IMI. Table 2 displays the means, standard deviations, and percentages of the different measures collected in the 7-day diary. Participants who completed the diary reported an average of 6.5 images in their diary, with a mean of 0.9 images per day.

**Table 2:** Pain-related intrusive mental image characteristics.

Total n of images	226 (90% recurring)
Mean images/week	6.5

Distribution of IMI frequency during the week	
0 IMI	2 (5.7%)
1 IMI	4 (11.4%)
2-5 IMI	12 (34.3%)
6-10 IMI	12 (34.3%)
11-15 IMI	0 (0.0%)
16-20 IMI	4 (11.4%)
>20 IMI	1 (2.9%)

Mean vividness (0-10)	6.5 (2.1 SD)
Mean postimage pain (0-10)	6.0 (1.8 SD)
Mean emotional intensity (0-10)	5.4 (2.6 SD)

**Predominant emotion associated to the image**	
Anger 78 (34.5%)	Other 75 (33.2%)
Sadness 28 (12.4%)	Frustration 14 (6.2%)
Fear 20 (8.8%)	Despair 5 (2.2%)
Calm 18 (8.0%)	Fed up 5 (2.2%)
Relief 5 (2.2%)	Indifference 5 (2.2%)

Valence of image (−10 to 10)		
Positive images (>0-10)	n = 21 (9.4%)	Mean valence +5.9 (3.0 SD)
Neutral images (value = 0)	n = 20 (9.0%)	Valence: 0
Negative images (−10 to <0)	n = 182 (81.6%)	Mean valence: −6.3 (2.2 SD)

Modality of image	Unimodal images	Multimodal images
	**n = 164** (72.6%)	**n = 55** (24.3%)
Visual	88 (53.7%)	48 (87.3%)
Auditory	14 (8.5%)	4 (7.3%)
Cutaneous	20 (12.2%)	20 (36.4%)
Kinesthetic	34 (20.7%)	38 (69.1%)
Olfactory	3 (1.8%)	1 (1.8%)
Gustatory	5 (3.0%)	2 (3.6%)

Image frequency reflects the total number of IMI recorded by each participant across the diary period. Missing values: valence was not reported for 3 images, predominant emotion was not reported for 2 images, and modality was not reported for 7 images.

Ninety percent of the images were described as recurring images. Most participants recorded between 2 and 10 images during the diary period (68.6%), with a few participants reporting no images (5.7%) or more than 20 images (2.9%). Twenty-six participants (74%) felt the sampled week was comparable to their normal average, while 4 participants (11%) felt they had more, and 4 participants (11%) felt that they had fewer images than usual, including the 2 participants who had no images during the sample week. One participant did not answer this question. Several participants explained during debriefing that they reported only a part of the experienced images, either because they forgot or felt it was too time consuming. When observing the data entry, it was noted that 11 participants reported only 1 occurrence per day, which could be the first occurrence of the IMI, or the only one. For these reasons, the frequency of pain-related IMI reported in this study is likely an underestimation of what patients really experienced.

Moreover, despite explicit requests to report images immediately upon experiencing them, some participants reported images only at the end of the day (n = 6), which might have been when they occurred, or a summary of the day.

The vividness of IMI (mean 6.5/10) and pain after the intrusion (mean 6.0/10) were moderate. The most frequent predominant emotion was anger (78/226 of images). The intensity of the predominant emotion (mean 5.4/10) was moderate. IMI were of negative valence in 81.6% of cases, positive in 9.4%, and neutral in 9.0%. Most of the images were in a single sensory modality, mainly visual (53.7%); 24.3% were multisensory.

Table 3 illustrates the trigger/context categories (multiple selections allowed) with examples. Many images were triggered by movement and/or pain, but most participants also mentioned other triggers/contexts, often citing waking up or resting. In addition, 7 images were reported to be triggered by stress or a particular emotion, like anger, and 9 occurred when feeling tired.

**Table 3:** List of triggers/contexts of the intrusive mental images reported in the diary, with examples.

Trigger/context	Example	N images
1. Pain	“Widespread pain”	84
2. Discussion or other external input	“While talking to a friend who will be hospitalized soon”	17
3. Movement/activity	“Physiotherapy exercise”	88
4. Memory	“Memory of radiotherapy”	17
5. Other		113
At rest/lying down	“While lying down, with no particular reason”	24
While sleeping/dreaming	“In a dream”	15
When waking up	“Upon waking up”	32
Emotion/stress	“Not sure if stress is a trigger, but that's how I feel right now”	7
Fatigue	“An intense fatigue”	9

During debriefing, most participants (80%) felt that the pain triggered the images, while 11% saw no relationship between the occurrence of the image and the pain, and 9% were unsure about a pain-image relationship. Among those who perceived pain to trigger IMI, 20% agreed that the images also contributed to the pain, 71% did not agree, and 9% were unsure.

Finally, time did not show any significant effect (*t* (31) = −0.40, *P* = 0.69, *d* = −0.07) on HADS-Total score (before M = 16.5 ± 6.4 SD; after M = 16.8 ± 6.1 SD), nor on its subscales HADS-A score (*t* (31) = 0.07, *P* = 0.95, *d* = 0.01; before M = 9.1 ± 3.4 SD; after M = 9.0 ± 3.3 SD) and HADS-D score (*t* (31) = −0.72, *P* = 0.48, *d* = −0.13; before M = 7.4 ± 4.1 SD; after M = 7.7 ± 3.8 SD), suggesting stability of the mood after keeping the diary.

### 3.3. Thematic categories of the intrusive mental images

Table 4 illustrates the thematic categories with examples of reported images. The number of images in each category reveals that IMI most frequently depicted the pain itself, followed by images related to the past. Notably, 11 participants reported 19 images that were described as coping images (ie, 31.4% of participants experiencing such images). Although most coping images were rated as positive, this was not always the case. For example, 1 patient reported a coping image to be associated with frustration and of negative valence, as the image failed to provide the usual relief. It was allowed to allocate each IMI to different categories (eg, an image of an injection on the forehead was categorized by the participant as a *pain-related memory* as well as a *coping image*). Only 1 image was classified as “other.” It was the image of having a hard piece of wood instead of the thigh. Since the participant described the image as accompanied by the sensation that it is not their leg, the participant felt the category “images of the pain itself” was not entirely appropriate. We propose for this image a new category depicting a depersonalization/dissociation phenomenon. This element was not included in the one-way ANOVAs described below.

**Table 4:** Examples of intrusive mental images from the 8 thematic categories.

Category	Examples	N images	Postimage pain	Emotional intensity	Valence
			M (SD)	M (SD)	M (SD)
1. The pain itself		113	6.0 (1.8)	5.1 (2.6)	−5.2 (3.2)
Anatomical representation	“Muscle is too short” “The muscles in my neck tighten and twist” (cutaneous)				
Metaphorical representation	“Trolls climbing up my spine with ice axes” “Like a ball of needles stabbing me with every breath” (cutaneous)				
2. Oneself in pain	“I see my body bent in pain” “Body is being torn apart” (kinesthetic)	25	6.3 (1.4)	5.5 (2.6)	−6.2 (2.5)
3. Social interactions in a pain context	“The doctor's face when he told me about the hole” “Body odor from sweat and medicinal odor” (olfactory)	7	6.9 (1.8)	5.0 (4.3)	−7.6 (2.7)
4. Projections about the future	“Daily life that will get more difficult” “The ground I'm walking on is made of spears” (cutaneous)	13	6.3 (2.3)	5.8 (2.4)	−4.6 (3.6)
5. Memory of a pain-related event	“Myself trying to get up after the accident” “An unpleasant, metallic taste” (gustatory)	40	5.3 (2.1)	5.9 (2.5)	−5.1 (3.8)
6. Coping images	“A big magnet attracting needles” “Very calm, relaxing music” (auditory)	19	6.0 (1.7)	5.9 (2.5)	+3.0 (6.0)
7. Negative self-appraisals	“Myself as a 90-year-old man” “I'm riding a motorcycle, but I can't use my foot to press the brake” (kinesthetic)	25	5.3 (2.1)	5.3 (3.2)	−6.3 (2.9)
8. Abstract image	“Blue like the sky” “A pulsating ball” (cutaneous and kinesthetic)	13	5.6 (1.1)	6.6 (1.7)	−1.5 (6.5)

The first example is always visual mental imagery; the second represents another sensory modality.

Postimage pain was assessed across image categories. No significant difference in mean pain intensity across categories was found (*F* (7,242) =  1.681, *P*  =  0.114, *η*^*2*^_*P*_  =  0.046). Exploratory analyses for emotional intensity and valence were performed across categories. No differences were observed for emotional intensity (*F* (7,42.7) =  1.422, *P*  =  0.222, *η*^*2*^_*P*_  =  0.031) either. However, results were significant for emotional valence (*F* (7,46.5) = 6.936, *P* < 0.001, *η*^*2*^_*P*_  =  0.304) with post hoc pairwise comparisons showing that coping images had a positive mean valence which differed significantly from all other categories (abstract images *P*  =  0.022; all others *P*  <  0.001). In addition, abstract images differed significantly in emotional valence from images of the pain itself (*P* =  0.028), oneself in pain (*P*  =  0.010), social interactions in the context of pain (*P*  =  0.017), and negative self-appraisals (*P*  <  0.01) (Table 4). No significant post hoc comparisons were found for projections about the future (*P* =  0.998) and memories of a pain-related event (*P*  =  0.099).

### 3.4. Correlations between baseline psychological measures and pain scores, intrusive mental image frequency, intrusive mental image characteristics, and postimage pain

HADS-Total and PCL-5 significantly correlated with BPI interference, whereas PCS and SUIS were significantly correlated with BPI severity (Table 5, Spearman test, uncorrected). IMI frequency in the diary did not significantly correlate with any of the assessed measures. Among IMI characteristics, emotional intensity correlated significantly with all measures, except for the SUIS and image frequency. Moreover, vividness significantly correlated with PCL-5 and both BPI measures. Valence correlated significantly negatively with PCL-5, both BPI measures, and PCS. Finally, postimage pain correlated significantly positively with PCS, both BPI scores, vividness, and emotional intensity and negatively with valence.

**Table 5:** Spearman correlations between psychological measures, image characteristics, and postimage pain.

	1. HADS-Total	2. PCS	3. PCL-5	4. SUIS	5. BPI severity	6. BPI interference	7. N images	8. Vividness	9. Emotional intensity	10. Valence	11. Postimage pain
1. HADS-Total	—	**0.45** *	**0.58** *	−0.06	0.08	**0.46** *	0.16	0.32	**0.55** *	−0.17	0.16
2. PCS		—	0.33	0.07	**0.34†**	0.31	−0.14	0.30	**0.62** *	**−0.56** *	**0.44†**
3. PCL-5			—	0.02	0.32	**0.56** *	0.32	**0.41†**	**0.63** *	**−0.36†**	0.33
4. SUIS				—	**0.41†**	0.08	0.09	0.19	−0.13	0.14	0.30
5. BPI severity					—	**0.56** *	−0.10	**0.41†**	**0.40†**	**−0.41†**	**0.69** *
6. BPI interference						—	0.27	**0.37†**	**0.47** *	**−0.41†**	**0.51** *
7. N images							—	0.04	−0.07	0.26	−0.11
8. Vividness								—	**0.66** *	−0.14	**0.64** *
9. Emotional intensity									—	**−0.35†**	**0.44†**
10. Valence										—	**−0.56†**
11. Postimage pain											—

Statistically significant *P*-values (*P* < 0.05) are shown in bold.

* *P* < 0.01 (uncorrected).

† *P* < 0.05.

HADS, Hospital Anxiety and Depression Scale; PCS, Pain Catastrophizing Scale; PCL-5, PTSD Checklist for DSM-5; SUIS, Spontaneous Use of Imagery Scale; BPI, Brief Pain Inventory.

### 3.5. Multilevel model analysis of baseline psychological measures and intrusive mental image characteristics predicting postimage pain

The intercept-only model indicated substantial between-person variability in pain ratings (ICC = 0.36), supporting the use of multilevel modelling.

All diary-level image characteristics were significantly associated with postimage pain, with higher vividness, greater emotional intensity, and more negative valence predicting higher pain ratings. Among the baseline psychological measures predictors, pain catastrophizing (PCS) was also significantly associated with higher ratings, whereas SUIS, HADS-Total, and PCL-5 were not significant predictors (Table 6).

**Table 6:** Multilevel model analysis predicting postimage pain.

Predictor	Estimate	SE	95% CI lower	95% CI upper	*t*	*P*	Partial *R*^2^
Intercept	2.747	1.240	0.317	5.177	2.215	0.034	NA
Vividness	0.187	0.068	0.053	0.320	2.744	**0.007**	0.014
Emotional intensity	0.199	0.056	0.089	0.309	3.546	**0.001**	0.023
Valence	−0.056	0.026	−0.107	−0.005	−2.148	**0.033**	0.009
SUIS	0.046	0.025	−0.003	0.095	1.830	0.078	0.029
HADS-Total	−0.065	0.050	−0.163	0.032	−1.318	0.198	0.028
PCS	0.055	0.020	0.015	0.096	2.711	**0.011**	0.060
PCL-5	0.040	0.021	0.000	0.081	1.945	0.062	0.066

Statistically significant *P*-values (*P* < 0.05) are shown in bold.

SUIS, Spontaneous Use of Imagery Scale; HADS, Hospital Anxiety and Depression Scale; PCS, Pain Catastrophizing Scale; PCL-5, PTSD Checklist for DSM-5.

Multicollinearity diagnostics indicated no problematic collinearity among predictors, with VIFs ranging from 1.15 to 2.83.

## 4. Discussion

This prospective diary study revealed a high prevalence (70%) of pain-related IMI in a population of patients with postsurgical or posttraumatic secondary chronic pain. Among the 226 reported IMI (mean 6.5 IMI/week), most were recurring, visual, and negatively valenced, with anger being the most frequently associated emotion. PTSD symptoms and baseline pain measures correlated with *qualitative IMI characteristics* (ie, vividness, emotional intensity, valence), but *not IMI frequency*. Furthermore, IMI vividness, emotional intensity, and valence were associated with postimage pain, whereas IMI frequency was not. In fact, multilevel modelling indicated that vividness, emotional intensity, and valence were significant predictors of postimage pain, with pain catastrophizing emerging as a significant baseline predictor.

The first aim was to compare participants with and without IMI. As shown before, people who regularly use mental imagery are more prone to experience IMI in chronic pain.^22^ Our data also confirmed that the presence of IMI was linked to higher depressive symptoms.^21–23,69^ As previously suggested, IMI could reinforce a vicious cycle: favoring emotional distress, avoidance, and hence depressive symptoms.^4,5,68^ In addition, despite excluding current PTSD, those with IMI reported higher posttraumatic symptoms, suggesting that IMI may be a common underlying factor. Interestingly, some unexplained chronic pain symptoms in the context of PTSD may be due to painful flashbacks^41^ (ie, re-experiencing painful sensations from the trauma). No differences were found between IMI presence and age, sex, symptoms of anxiety, catastrophizing, and pain scores. Previous research found no differences for age and sex either.^22^ By contrast, several studies^21–23,69^ did find differences between IMI presence and anxious symptoms as well as catastrophizing. This may be due to a negative recall bias in anxious or catastrophizing individuals in retrospective designs. Finally, consistent with prior research,^23^ no differences between groups regarding pain *duration* were found. Graham et al.^23^ reported higher pain *intensity* in participants with IMI, while Gosden et al.^22^ did not, aligning with our results. Finally, overall pain and distress levels were slightly lower than in prior IMI patient studies,^5,21,69^ yet consistent with other studies of secondary chronic pain.^34^

The second aim was to examine whether IMI predicted postimage pain, and we highlighted that IMI characteristics interacted with pain following imagery episodes. This aligns with previous research showing that emotionally charged IMI can exacerbate affective and sensory pain components^5,50^ and with recent EMA research linking imagery characteristics to pain outcomes.^67^ These authors also identified image frequency and trait imagery (SUIS) as predictors of pain outcomes. These factors did not correlate with postimage pain in this study. Nevertheless, catastrophizing was a separate predictor of postimage pain, highlighting its robust role in modulating the pain experience and relevance for future imagery research, consistent with fear-avoidance models of chronic pain.^59^ Similarly, prior work suggests that how IMI are interpreted or experienced may be more relevant than their occurrence: Watts et al.^69^ found that negative interpretations of IMI predicted pain disability but not their mere presence, whereas Gosden et al.^22^ found image frequency to be related to pain unpleasantness but not pain intensity. However, such associations should be interpreted with caution, as pain and mood before IMI onset were not assessed and may influence IMI characteristics as well as postimage pain.

Given heterogeneity in the literature, it is worth briefly discussing the IMI characteristics found in this study. Our study was designed to minimize biases in estimating the image prevalence and frequency. The initial interview reduced a potential inclusion bias. Potential confusions with pain metaphors^2,27,28^ were prevented by: ensuring a clear definition of IMI during the first interview; keeping the diary outside of interpersonal contexts; verifying IMI descriptions in the last interview. The prospective electronic diary allowed immediate reporting of pain-related IMI, reducing memory biases. Diary-keeping could have triggered images (potential provocation bias), but the perceived representativity of the sampled week was confirmed for most participants. The identified prevalence is on the higher side compared with prior reports in populations with chronic pain (23%-100%).^5,21–23,51,66,69^ Our reported frequency (mean 6.5 IMI/week) aligns with IMI diary studies in different populations, for example, in PTSD, where trauma-related intrusions ranged from 7.3^38^ to 7.7^63^ per week but is higher than in other pain-IMI studies, where only 18% to 24% of participants reported daily experiences.^23,69^ IMI total number ranged from 0 to 24 (2 participants reporting no IMI), aligning with diary studies outside the pain context (eg, in 46 trauma survivors, 0-41 IMI/week, 2 reporting none^38^).

Participants reported IMI fitting all 8 thematic categories (based on previous work).^2,5,22,51,72^ Of note, a recent qualitative study organized IMI into only 3 key categories, which may be due to the small sample size (n = 10) not capturing the full range of potential content.^17^ Although our results provide support for the existing IMI categories, future qualitative work with larger samples and participant involvement may help further refine them and evaluate their usability. Coping images were as frequent as in a prior IMI study in endometriosis (30%)^23^ and were characterized by a positive emotional valence. Although not associated with reduced postimage pain, they might have an influence on the affective component of pain (ie, pain unpleasantness). This hypothesis could be explored in further research. The moderate average vividness (mean 6.5/10) was in line with previous pain-related IMI reports,^22^ while vividness may be higher in trauma-related IMI.^6,43^ Previous studies have focused on visual imagery.^21,51^ Our findings confirm a predominance of visual imagery, yet demonstrate images in all sensory modalities. These could deserve specific future investigations. Nighttime (sleeping, dreaming), waking up, and being at rest were frequently reported triggers/contexts despite not being on the suggested list. Hence, IMI may be favored by physical stillness or transitions between activity states, potentially reflecting heightened cognitive or sensory processing in these moments. In fact, significant pain during nighttime was shown to intrude consciousness in the form of pain-related IMI while dreaming.^17^ More refined methods (eg, in-depth qualitative analyses) could be needed to fully explore context and triggers.

Finally, IMI might hold therapeutic potential. Interestingly, some participants reported trying to modify their IMI. For example, the patient reporting the IMI of trolls climbing up her spine with ice axes voluntarily imagined negotiating with the troll leader. Other participants also spontaneously expressed hope that addressing IMI by means of journaling could be beneficial. Yet stable HADS scores after journaling suggest that it is neither sufficient to improve psychological measures nor counterproductive. It has been proposed in the CBT literature to identify and address maladaptive mental images,^4,5^ although this remains little studied. In fact, widely used and studied CBT approaches for pain, such as acceptance and commitment therapy, mostly target maladaptive verbal thoughts.^71^ Nevertheless, (1) experimental studies have targeted mental imagery with an impact on pain perception^3,50^ and (2) clinical studies suggest benefits of interventions addressing IMI, such as imagery rescripting,^31,40^ guided imagery,^36,53,54^ and hypnosis.^14,65,73^ IMI could also be targeted with cognitive retraining approaches used in nonpain fields, for example, cognitive bias modification,^8,9^ metacognitive approach,^47,70^ or mindfulness-based cognitive therapy.^29,62^ Accordingly, it may be useful to more routinely assess IMI, their context and associated emotions in clinical and research settings and orient patients reporting negative IMI towards specific imagery-based therapeutic interventions.

### 4.1. Limitations and future research

This study was not preregistered. Some participants did not comply with the instructions and either reported only the first occurrence of each IMI or all IMI at the end of the day, leading to a possible underestimation of IMI frequency. Although this prospective diary methodology is an improvement over retrospective accounts, it is burdensome. Future technology may ease this while enhancing accuracy (eg, by incorporating brief daily checks to capture unreported images).

There is an important range of reported images among participants (0-24), with those reporting more images exerting a greater influence on some results. Furthermore, journal keeping was more taxing for participants with many images, possibly introducing a selection bias. However, their interest in exploring the phenomenon may have increased their motivation to participate.

A list of associated emotions was provided, including the most frequently cited emotion, anger. Interestingly, frustration was mentioned in 6% of images, despite not being suggested, and might therefore have been under-reported. Hence, frustration should be included as a predefined emotion in future research. Finally, IMI intrusiveness was not reliably assessed and should be captured in the future.

### 4.2. Conclusion

Pain-related IMI are prevalent, occur frequently, and tend to be recurrent. Vividness, emotional intensity, and valence were significant predictors of postimage pain, alongside baseline catastrophizing. Hence, IMI characteristics interact with the pain experience. This study deepens the understanding of intrusive mental images as a relevant cognitive process that can be part of the pain experience, potentially informing targeted therapeutic interventions.

## Conflict of interest statement

The authors have no conflict of interest to declare.

## Supplementary Material

## Appendix A. Supplemental digital content

Supplemental digital content associated with this article can be found online at http://links.lww.com/PAIN/C505.
