# Supplementary material for: A prospective diary study of chronic pain-related intrusive mental imagery: “trolls climbing up my spine with ice axes” and other vivid images
Source: Pain. 2026 May 29;167(8):e332–43. doi: 10.1097/j.pain.0000000000004009 (PMC13382862; doi:10.1097/j.pain.0000000000004009)
Supplement: Supplementary file 1 [file jop-167-e332-s001.pdf]

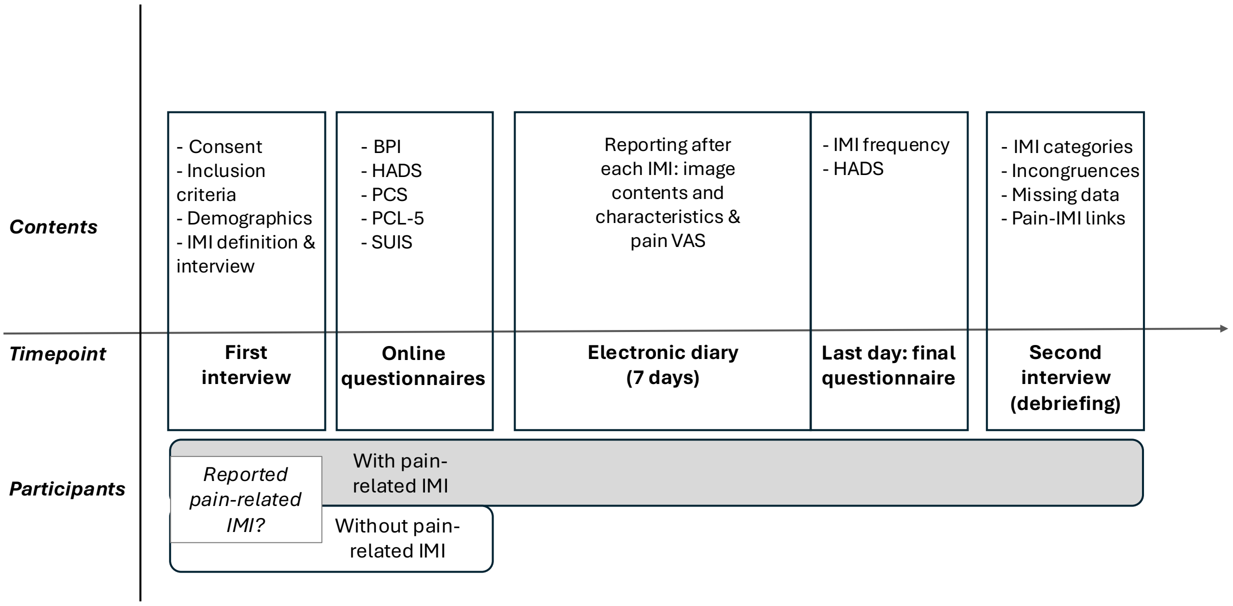


**Supplementary Figure 1: Overview of the study procedure**

**Supplementary Table 1**

**Diary instructions and measures**

| Item | Question/Instruction | Response format |
|---|---|---|
| Title | Intrusive mental imagery diary – New intrusive mental image | – |
| Identification | Personal code: | Code |
| 1. Content | Please describe the intrusive mental image in a few words: | Open text |
| 2. Recurrence | Is this image new or recurrent? | Categorical single choice: New image or Recurrent image |
| 3. Context/Trigger | At what moment did it appear? Did you notice a trigger? Please describe the context in which it appeared in a few words. Specific Trigger (suggested options pain, discussion, movement, memory, other)? | Open text |
| 4. Intrusiveness | How much did you intend to have this image? | VAS (0 – 10); anchors: No intention – Image voluntarily evoked |
| 5. Vividness | How vivid was the image? | VAS (0 – 10); anchors: Not vivid – Extremely vivid |
| 6. Associated emotion | Please select the predominant emotion experienced: | Categorical single choice: Sadness, Fear, Anger, Relief, Calm, Other (specify, open text) |
| Emotional intensity | Please rate the intensity of the predominant emotion experienced: | VAS (0 – 10); anchors: No emotion felt – Extremely strong |
| Emotional valence | Please rate the valence of the predominant emotion experienced: | VAS (-10 – 10); anchors: Extremely unpleasant – Extremely pleasant |
| 7. Sensory modality | Please select the sensory modality or modalities in which this image was experienced from the following list: | Categorical with multiple choices: Visual, Auditory, Gustatory, Olfactory, Cutaneous (touch / sensation of being touched), Kinesthetic (movement / action) |
| 8. Post-image pain | Please rate the pain you are experiencing now: | VAS (0 – 10); anchors: No pain – Worst pain imaginable |

VAS = Visual Analog Scale
Please note that the original participant instructions were provided in French. For broader accessibility and understanding, the instructions are reported in English.
